# Metformin regulates chondrocyte senescence and proliferation through microRNA-34a/SIRT1 pathway in osteoarthritis

**DOI:** 10.1186/s13018-023-03571-5

**Published:** 2023-03-13

**Authors:** Shiju Yan, Wenjing Dong, Zhirui Li, Junqiang Wei, Tao Han, Junliang Wang, Feng Lin

**Affiliations:** 1 Department of Orthopedics, Hainan Hospital of Chinese PLA General Hospital, 80 Jianglin Road, Sanya, Hainan People’s Republic of China; 2 Department of Gerontology, Hainan Hospital of Chinese PLA General Hospital, Sanya, People’s Republic of China

**Keywords:** Metformin, MicroRNA-34a, SIRT1, Senescence, Proliferation, Osteoarthritis

## Abstract

**Background:**

Osteoarthritis (OA) is the most common degenerative disease in joints among elderly patients. Senescence is deeply involved in the pathogenesis of osteoarthritis. Metformin is widely used as the first-line drug for Type 2 diabetes mellitus (T2DM), and has great potential for the treatment of other aging-related disorders, including OA. However, the role of metformin in OA is not fully elucidated. Therefore, our aim here was to investigate the effects of metformin on human chondrocytes.

**Methods:**

After metformin treatment, expression level of microRNA-34a and SIRT1 in chondrocyte were detected with quantitative real-time PCR and immunofluorescence staining. Then, microRNA-34a mimic and small interfering RNA (siRNA) against SIRT1 (siRNA-SIRT1) were transfected into chondrocyte. Senescence-associated β-galactosidase (SA-β-gal) staining was performed to assess chondrocyte senescence. Chondrocyte viability was illustrated with MTT and colony formation assays. Western blot was conducted to detect the expression of P16, IL-6, matrix metalloproteinase-13 (MMP-13), Collagen type II (COL2A1) and Aggrecan (ACAN).

**Results:**

We found that metformin treatment (1 mM) inhibited microRNA-34a while promoted SIRT1 expression in OA chondrocytes. Both miR-34a mimics and siRNA against SIRT1 inhibited SIRT1 expression in chondrocytes. SA-β-gal staining assay confirmed that metformin reduced SA-β-gal-positive rate of chondrocytes, while transfection with miR-34a mimics or siRNA-SIRT1 reversed it. MTT assay and colony formation assay showed that metformin accelerated chondrocyte proliferation, while miR-34a mimics or siRNA-SIRT1 weakened this effect. Furthermore, results from western blot demonstrated that metformin suppressed expression of senescence-associated protein P16, proinflammatory cytokine IL-6 and catabolic gene MMP-13 while elevated expression of anabolic proteins such as Collagen type II and Aggrecan, which could be attenuated by transfection with miR-34a mimics.

**Conclusion:**

Overall, our data suggest that metformin regulates chondrocyte senescence and proliferation through microRNA-34a/SIRT1 pathway, indicating it could be a novel strategy for OA treatment.

## Introduction

Osteoarthritis (OA) is a prevalent age-related joint disorder characterized by progressive cartilage destruction, synovitis, subchondral bone remodeling and osteophyte formation [1]. OA causes pain and joint dysfunctions in the affected patients, leading to massive burden on social health care system. Various factors, including age, obesity, trauma history, abnormal mechanical stimulation, microRNAs and genetic predisposition, are closely related with both onset and progression of OA [2, 3]. However, there are no effective disease-modifying treatments that could reverse or delay OA development due to specific mechanisms leading to OA have not been fully elucidated. And more often, surgical treatments are essential for most patients with late-stage OA to improve life quality.

At present, etiology and pathogenesis of OA are complex and still remain not completely understood. Chondrocyte, the only cell type present in articular cartilage, plays a vital role in maintaining the dynamic homeostasis of anabolism and catabolism of the cartilage extracellular matrix (ECM) [4]. It is well accepted that chondrocyte senescence has been noted as a major event contributing to age-related changes in cartilage homeostasis, integrity and physiological function [5]. However, the exact mechanism linking senescence with OA pathogenesis remains to be further investigated and a better understanding of mechanisms of senescence underlying OA could provide new therapeutic targets for OA prevention and treatment.

Metformin has been widely used as the first-line medication for Type 2 diabetes mellitus (T2DM) by activating adenosine monophosphate-activated protein kinase (AMPK) [6], which lay a solid foundation for metformin to regulate metabolic and cellular processes, such as senescence, inflammation, oxidative stress and apoptosis [7, 8]. Evidence suggests that metformin has high safety profile and could be promising for a number of age-related diseases, such as degenerative musculoskeletal diseases, cardiovascular diseases and neurodegenerative diseases [9]. Recent study has been discussing the potential use of metformin in OA management. Metformin attenuates cartilage degradation and modulates pain-related behavior in a destabilization of the medial meniscus (DMM) model of OA in mice [10]. Latest research shows that metformin treatment decreases cartilage degradation, inhibits cartilage matrix catabolism and enhances cartilage matrix anabolism in both the early and late stages of OA [11]. Thus, metformin could be prospective in OA treatment.

MicroRNAs are a family of endogenous non-coding RNAs with 18 ~ 24 nucleotides in length [12]. MicroRNAs could regulate gene expression at post-translational level by binding to the 3′-untranslated region (3′-UTR) of target mRNAs [13]. Numerous studies have shown that microRNAs are involved with various cellular processes, such as senescence, cell proliferation, apoptosis and migration [3, 12, 14]. MicroRNA-34a (miR-34a) has been reported as a potential indicator of senescence, since its expression is increased in several aging tissue and cells, including chondrocytes [15–17]. Moreover, microRNA-34a is a target gene of metformin, which regulates multiple biological processes by regulating the expression of microRNA-34a [18, 19]. Therefore, exploring the roles of microRNAs and their potential target genes is critical for understanding the molecular mechanisms of OA pathogenesis.

Sirtuins (silent information regulator proteins) are a family of nicotinamide dinucleotide (NAD^+^)-dependent deacylases and highly conserved from bacteria to humans, which control a variety of cellular processes, including DNA repair, cell cycle, mitochondria homeostasis and cellular senescence [20, 21]. SIRT1 is the best studied sirtuins in bone and cartilage and is critical in maintaining cartilage health by promoting chondrocyte survival and ECM homeostasis [22]. Furthermore, evidence suggests that SIRT1 is one of the direct targets of microRNA-34a and microRNA-34a promotes chondrocyte apoptosis by regulating SIRT1, contributing to OA pathogenesis [17, 23]. Thus, this study is aimed to explore the effects of metformin in OA, specifically on chondrocyte senescence and proliferation, and investigate the molecular mechanisms of it by focusing on its target gene microRNA-34a and the downstream microRNA-34a/SIRT1 signaling pathway.

## Materials and methods

### Patient and cohort description

This study was approved by Ethics Committee of the Hainan Hospital of Chinese PLA General Hospital (approval number: S2022-10), and informed consent was obtained from all patients. OA articular cartilage samples were aseptically harvested from femoral condyles and tibial plateaus of 14 patients diagnosed with osteoarthritis with a Kellgren/Lawrence (K/L) grade of 3 or 4 who received total knee arthroplasty (mean age ± SD: 69.6 ± 3.1 years) at Hainan Hospital of Chinese PLA General Hospital from January 2020 to December 2020. Healthy cartilage samples were obtained from 12 trauma amputees with no history of OA or other musculoskeletal diseases (38.6 ± 6.7 years).

### Isolation and cell culture of primary chondrocytes

Cartilage samples were minced into small pieces and then digested with 0.25% trypsin (Invitrogen, Carlsbad, USA) for 30 min and 0.2% collagenase Type II (Millipore, Billerica, USA) for 10 h in a shaking water bath at 37 °C. Isolated chondrocytes were filtered through 100 μm nylon filters, washed twice with sterile PBS and seeded into culture flasks in DMEM (Gibco, NY, USA) medium supplemented with 10% FBS (Hyclone, Thermo Scientific, USA), 100 U/mL penicillin and 100 μg/mL streptomycin (Gibco) at 37 °C in a humidified atmosphere with 5% CO_2_. Primary chondrocytes and cells from passage 1 were used for this serial experiments.

### Cell treatments and transfection

miR-34a mimic, negative control (NC) and siRNA against SIRT1 (siRNA-SIRT1) were designed and synthesized by RiboBio (Guangzhou, China). At 80% confluence, chondrocytes were treated with 1 mM metformin (Sigma-Aldrich, USA) for 48 h. After metformin treatment, chondrocytes were transfected with miR-34a mimics, siRNA against SIRT1 or negative control (NC), respectively, at working concentration of 100 nM for 48 h using Lipofectamine 2000 (Life technologies, USA) according to the manufacturer's protocol.

### Immunofluorescence staining

Metformin-treated chondrocytes were seeded into on 15 mm cell slides (Nest Biotechnology) in 6-well plates. Cells were fixed with 4% paraformaldehyde (Beyotime, China) for 15 min and permeabilized with 0.1% Trion X-100 in PBS for 20 min. Chondrocytes were blocked with 10% BSA for 30 min and then incubated with anti-SIRT1 primary antibody (1:100, #8469, Cell Signaling Tech) overnight at 4 °C. Subsequently, cells were washed and incubated with Alexa Fluor 555-conjugated secondary antibody (1:500, A32727, Invitrogen) for 1 h at room temperature. Finally, nuclei were stained with DAPI in the dark, and fluorescence images were obtained using Fluorescence microscope (IX70, Olympus, Japan).

### RNA extraction and real-time quantitative RT-PCR

Total RNA was extracted from chondrocytes using TRIzol reagent (Invitrogen, CA) according to the manufacturer's instructions. Total RNA was reverse transcribed with PrimeScript RT Master Mix (TAKARA, Japan). Quantitative Real-Time PCR was performed using Power SYBR Green PCR Master Mix (Applied Biosystems, Foster city, CA, USA) according to manufacturer's protocol. Specific primers used for mRNAs were listed below: for SIRT1, 5′- TGGCAAAGGAGCAGATTAGTAGG -3′ (forward) and 5′- CTGCCACAAGAACTAGAGGATAAGA -3′ (reverse); for GAPDH, 5′-ATTCCACCCATGGCAAATTC-3′ (forward) and 5′-TGGGATTTCCATTGATGACAAG-3′ (reverse) (Table 1). GAPDH mRNA was used as a housekeeping gene for normalization with the comparative 2^−ΔΔCt^ method.

**Table 1:** Primer sequence

Gene	Primer sequence (5′-3′)
SIRT1	Forward:TGGCAAAGGAGCAGATTAGTAGG
	Reverse:CTGCCACAAGAACTAGAGGATAAGA
microrna-34a	Forward:CGTCACCTCTTAGGCTTGGA
	Reverse:CATTGGTGTCGTTGTGCT
GAPDH	Forward:ATTCCACCCATGGCAAATTC
	Reverse:TGGGATTTCCATTGATGACAAG
U6	Forward:CTCGCTTCGGCAGCACATATACT
	Reverse:ACGCTTCACGAATTTGCGTGTC

For relative quantification of microRNA-34a, RNA was reverse transcribed using the TaqMan MicroRNA Reverse Transcription kit (Applied Biosystems, USA) according to the manufacturer’s protocol and Real-Time quantitative-PCR was performed with the TaqMan MicroRNA assays (Applied Biosystems, USA). The reactions were performed in 7500 Fast Real-Time System (Applied Biosystems, USA). Small nuclear RNA U6 was used as housekeeping gene for normalization (Table 1).

### SA-β-galactosidase staining

48 h after transfection, OA chondrocytes were fixed and stained with Senescence β-Galactosidase Staining Kit (#9860, Cell Signaling Technologies, USA) according to the manufacturer’s protocol. Senescent cells were identified by formation of blue precipitates at pH 6 and counted from three different fields of view under microscopy. Percentage of SA-β-gal-positive chondrocytes were calculated.

### MTT assay

Cell proliferation was evaluated with MTT assay. 24 h after transfection, chondrocytes were seeded into 96-well plates at a density of 5 × 10^3^/well. 20 µl of MTT (5 mg/ml) (Sigma-Aldrich, USA) was added to each well every 24 h, followed by incubation at 37 °C for another 4 h. Culture medium was removed, and formazan precipitate was dissolved with 100 µl of dimethyl sulfoxide for 20 min at 37 °C. The optical density (OD) was determined by measuring the absorbance at 490 nm with a reference wavelength of 630 nm using a microplate reader (ThermoFisher, USA).

### Colony formation assay

Cell viability was evaluated with Colony formation assay. Transfected chondrocytes were seeded into 6-well plates (300/well) and cultured for 14 days in DMEM medium containing 10% FBS. Colonies were fixed with methanol for 20 min and stained with crystal violet (KeyGen, China) for 20 min at room temperature. Cell colony was observed under microscope (Olympus, Japan) and counted.

### Western blot analysis

48 h after transfection, chondrocytes were rinsed with PBS and treated with RIPA lysis buffer (Beyotime, China) containing enzyme inhibitor cocktail (Roche, Switzerland) on ice for 30 min. Protein concentrations were quantified using BCA protein assay kit (Beyotime, China). 25 μg of total protein for each sample was loaded and separated by 10% sodium dodecyl sulfate–polyacrylamide gel electrophoresis (SDS-PAGE) and transferred to PVDF membranes (Millipore, USA). Membranes were blocked with 5% skim milk and then incubated with primary antibodies against P16 (1:1000, ab51243, Abcam), ACAN (1:500, ab3778, Abcam), MMP13 (1:1000, #69,926, CST), IL-6 (1:1000, 66,146-1-Ig, Proteintech), COL2A1 (1:1000, ab34712, Abcam), SIRT1 (1:1000, #8469, CST), GAPDH (1:1000, ab9485, Abcam) overnight at 4 °C. Then, second antibodies were incubated for 2 h at room temperature after membranes were washed with TBST. Signals obtained with the ECL luminescent reagent (Millipore, USA) were detected and analyzed using ChemiDoc Imaging System (Bio-Rad, USA) with Quantity One analyzing system.

### Statistical analysis

Continuous variables were presented as mean ± standard deviation (SD). Comparisons between two groups were analyzed by unpaired Student's t test or Mann–Whitney U test. One-way analysis of variance (ANOVA) followed by post hoc LSD test was used for difference statistical analysis between multiple groups. All testing was performed using SPSS26.0 (IBM, USA). A difference with *P* < 0.05 was considered to be significant.

## Results

### Metformin regulated microRNA-34a and SIRT1 expression in chondrocytes

**Fig. 1:**
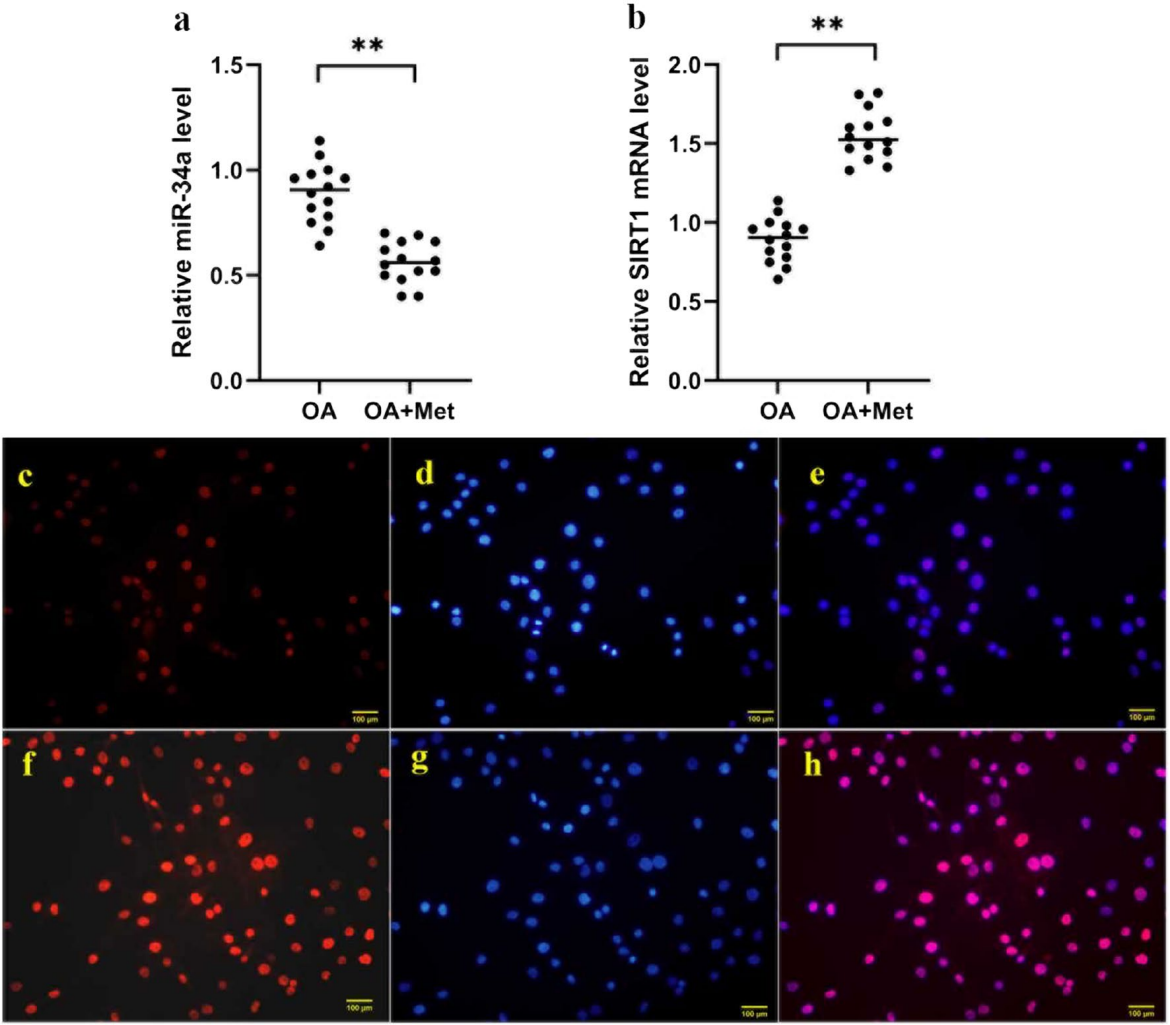
Metformin regulated microRNA-34a and SIRT1 expression in chondrocytes. OA chondrocytes were treated with 1 mM metformin. Metformin downregulated expression level of microRNA-34a (**a**) and promoted mRNA level of SIRT1 (**b**) in OA chondrocytes. (**c**)–(**h**) immunofluorescence staining for SIRT1. (**c**) SIRT1, (**d**) nuclei stained with DAPI and (**e**) merged field for chondrocytes in control group. (**f**) SIRT1, (**g**) nuclei stained with DAPI and (**h**) merged field for metformin-treated chondrocytes in Met group. All experiments were repeated three times. **P < 0.01

To investigate the mechanism and effects of metformin on OA, OA chondrocytes were treated with 1 mM metformin. As revealed in Fig. 1, metformin treatment significantly inhibited expression level of microRNA-34a and promoted mRNA level of SIRT1 in OA chondrocytes (*P* < 0.01).

As consistently shown in immunofluorescence staining (Fig. 1c–h), SIRT1 mainly located in the nuclei and its expression level was promoted (*P* < 0.05) by metformin treatment. Collectively, these results suggested microRNA-34a and SIRT1 were regulated by metformin in OA chondrocytes and could be related with biofunction of metformin.

### MicroRNA-34a regulated SIRT1 expression in chondrocytes

**Fig. 2:**
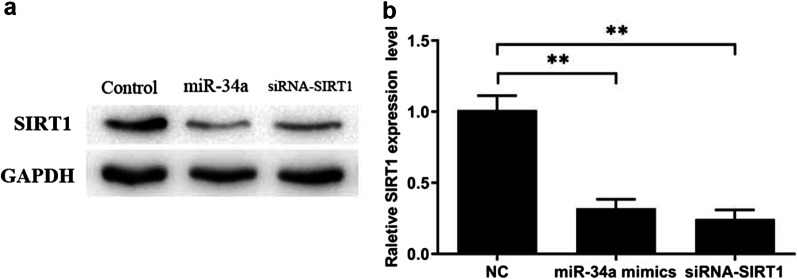
MicroRNA-34a regulated SIRT1 expression in chondrocytes. Chondrocytes were transfected with miR-34a mimics, siRNA against SIRT1 (si-SIRT1) or negative control (NC) at a working concentration of 100 nM, respectively. (**a**)–(**b**) miR-34a mimics and siRNA-SIRT1 inhibited SIRT1 expression in chondrocytes. All experiments were repeated three times. **P < 0.01

Chondrocytes were transfected with miR-34a mimics, siRNA against SIRT1 or negative control (NC), respectively. Western blot assay was performed to evaluate SIRT1 expression. Results demonstrated that both miR-34a mimics and siRNA against SIRT1 inhibited SIRT1 in chondrocytes, as revealed in Fig. 2.

### Metformin attenuated chondrocytes senescence

**Fig. 3:**
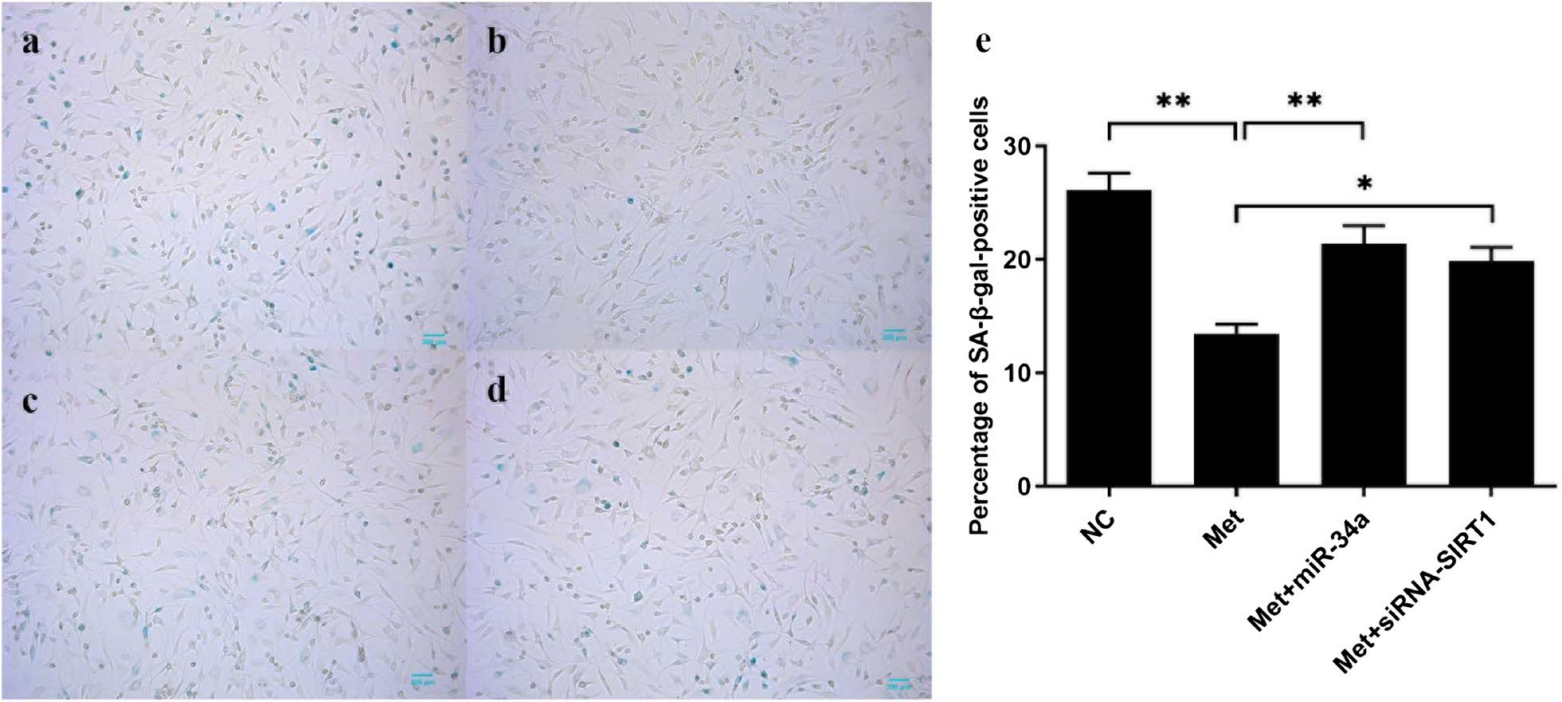
Metformin attenuated OA chondrocytes senescence in vitro. Chondrocytes were divided into Metformin treatment group (Metformin), Metformin + miR-34a group (Met + miR-34a), Negative Control group (NC) and Metformin + siRNA-SIRT1 group (Met + si-SIRT1). After treated with 1 mM metformin for 48 h, chondrocytes were transfected with 100 nM miR-34a negative control (**b**), miR-34a mimics (**c**) or siRNA-SIRT1 (**d**) for 48 h, respectively. NC group (**a**) was treated with culture medium as blank control. Percentage of SA-β-gal-positive chondrocytes were calculated. (**e**) metformin attenuated OA chondrocytes senescence and this effect was reversed by transfection of microRNA-34a mimics or siRNA against SIRT1. All experiments were repeated three times. *P < 0.05. **P < 0.01

We performed SA-β-galactosidase staining to evaluate the effects of metformin on chondrocyte senescence. Compared with NC group (Fig. 3a), metformin treatment significantly decreased percentage of SA-β-gal-positive OA chondrocytes in Metformin group (Fig. 3b). Importantly, this decrease was reversed by transfection of microRNA-34a mimics in Met + miR-34a group (Fig. 3c) or siRNA against SIRT1 in Met + si-SIRT1 group (Fig. 3d). These results indicated that metformin regulated chondrocytes senescence in vitro via microRNA-34a and SIRT1.

### Metformin promoted cell proliferation and viability of chondrocytes

**Fig. 4:**
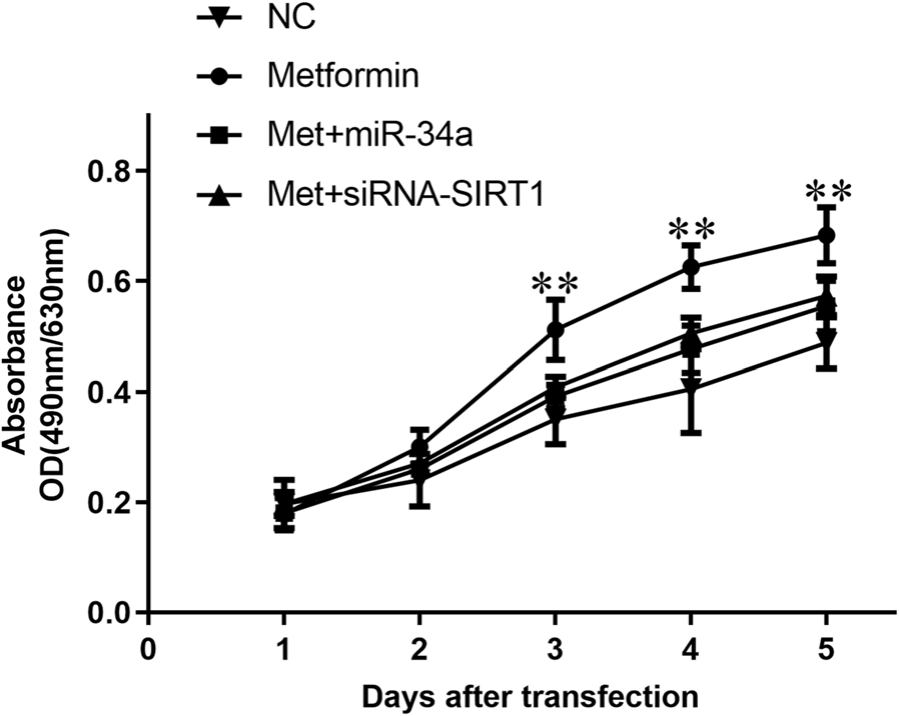
Metformin promoted chondrocytes proliferation in vitro. After treated with 1 mM metformin for 48 h, chondrocytes were transfected with 100 nM miR-34a negative control, miR-34a mimics or siRNA-SIRT1 for 48 h, respectively. NC group was treated with culture medium as blank control. 24 h after transfection, chondrocytes were seeded into 96-well plates and cell number was evaluated as the absorbance at 490 nm with a reference wavelength of 630 nm. All experiments were repeated three times. **P < 0.01

Next, MTT assay was performed and proliferation curve was recorded to explore the effects of metformin on proliferation of human chondrocytes. Figure 4 shows that chondrocytes incubated with metformin in Metformin group exhibited a significant increase in proliferation capacity compared with chondrocytes in the NC group. Moreover, when transfected with microRNA-34a mimics or siRNA against SIRT1 after metformin treatment, chondrocytes in Met + miR-34a group and in Met + si-SIRT1 group grew at a lower rate than chondrocytes in Metformin group. No statistical significance was observed in proliferation rate among NC group, Met + miR-34a group and Met + si-SIRT1 group (Fig. 4).

**Fig. 5:**
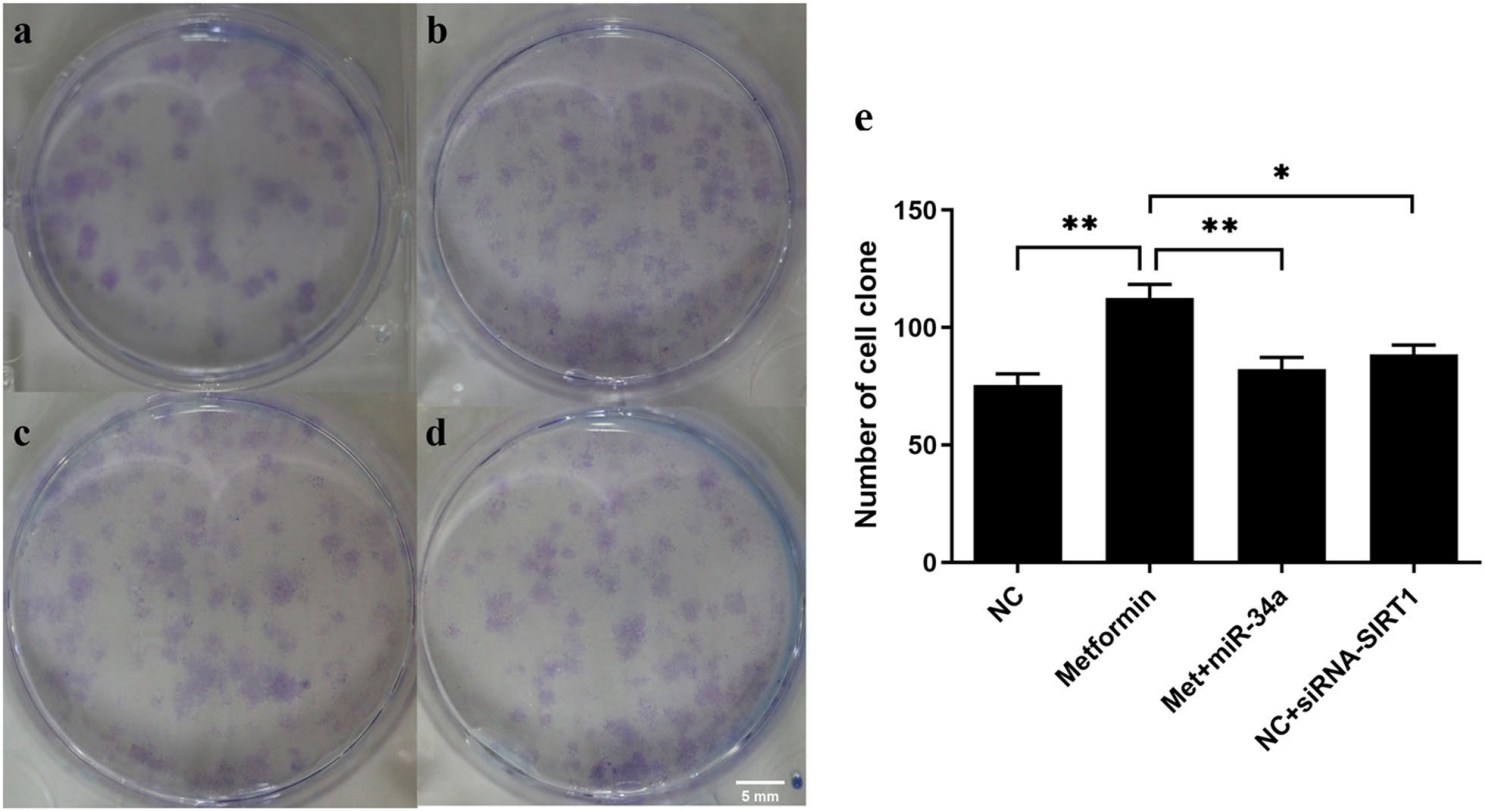
Metformin increased chondrocytes colony formation in vitro. After treated with 1 mM metformin for 48 h, chondrocytes were transfected with 100 nM miR-34a negative control (**b**), miR-34a mimics (**c**) or siRNA-SIRT1 (**d**) for 48 h, respectively. NC group (**a**) was treated with culture medium as blank control. Cell colony was observed and counted. (**e**) Chondrocytes treated with metformin in Metformin group formed increased cell colonies compared with NC group, while transfection of miR-34a mimic or siRNA for SIRT1 following metformin treatment inhibited cell colony formation in Met + miR-34a group and Met + si-SIRT1 group. All experiments were repeated three times. *P < 0.05. **P < 0.01

To further test the effect of metformin on chondrocyte viability, we performed colony formation assay. Chondrocytes treated with metformin in Metformin group (Fig. 5b) formed increased cell colonies compared with NC group (Fig. 5a), while transfection of microRNA-34a mimic or siRNA against SIRT1 following metformin treatment inhibited cell colony formation in Met + miR-34a group (Fig. 5c) or Met + si-SIRT1 group (Fig. 5d).

### Metformin ameliorated chondrocytes senescence and OA through microRNA-34a/SIRT1 pathway

**Fig. 6:**
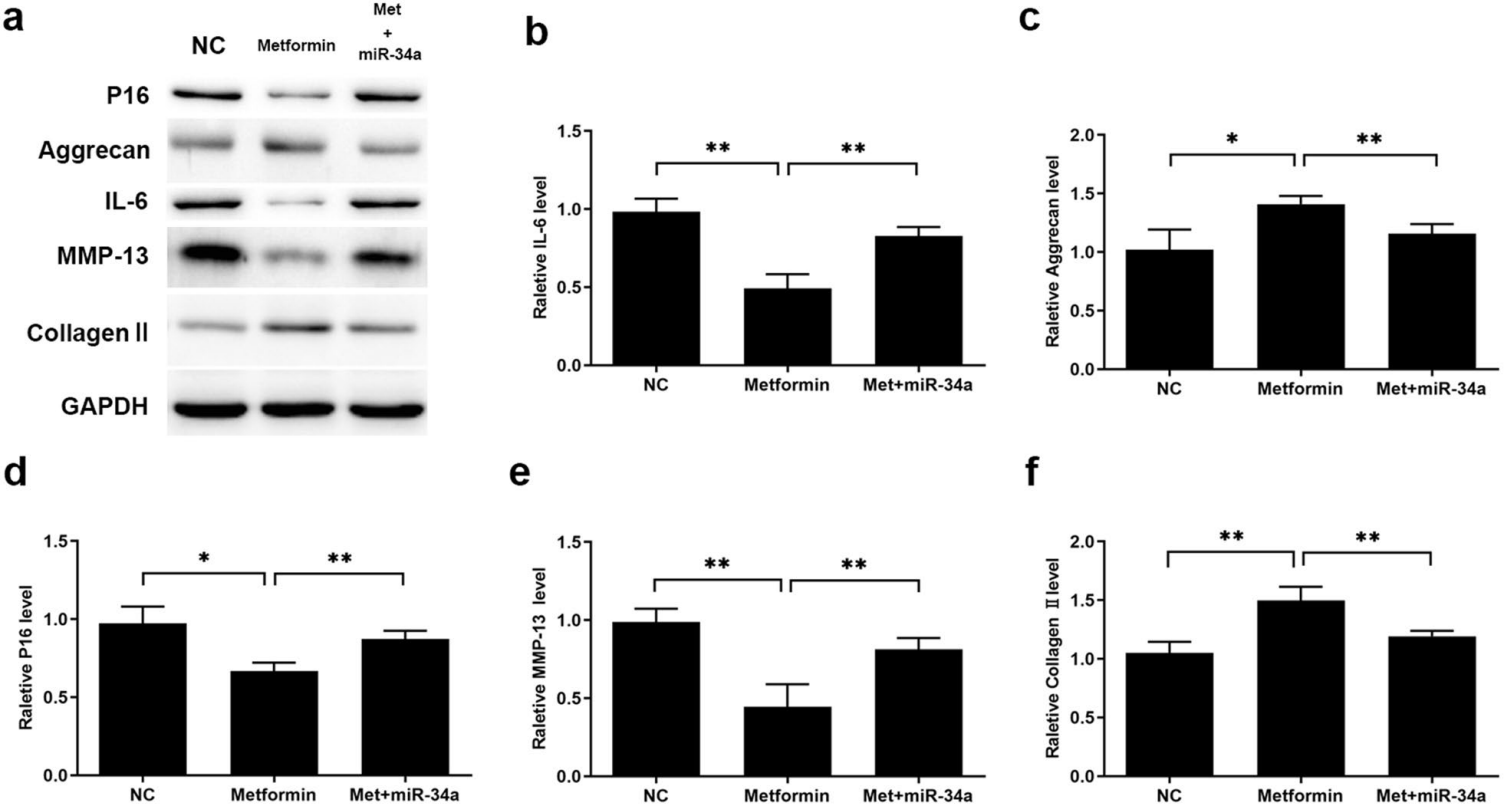
Metformin ameliorated chondrocytes senescence and OA through microRNA-34a/SIRT1 pathway. After treated with 1 mM metformin for 48 h, chondrocytes were transfected with 100 nM miR-34a negative control and miR-34a mimics for 48 h, respectively. NC group was treated with culture medium as blank control. Metformin treatment significantly inhibited P16 (**b**) expression in Metformin group compared with NC group. Furthermore, metformin promoted expression level of Collagen II (**f**) and Aggrecan (**c**) while downregulated expression level of MMP-13 (**e**) and IL-6 (**d**). Transfection of microRNA-34a mimic after metformin treatment reversed this favorable effect by increasing expression level of P16, IL-6 and MMP-13 while decreasing expression level of Collagen II and Aggrecan (**a**). All experiments were repeated three times. *P < 0.05. **P < 0.01

Western blot was performed to investigate the mechanism of metformin on senescence and metabolism homeostasis of chondrocytes. Results from western blot assay demonstrated that metformin treatment significantly inhibited P16 expression in Met group compared with NC group. Furthermore, metformin promoted expression level of COL2A1 (collagen II) and ACAN (aggrecan) while MMP-13, which regulates cartilage matrix degradation, and IL-6 were downregulated, suggesting that metformin cast a protective effect on chondrocytes against OA. On the contrary, microRNA-34a mimic transfection after metformin treatment counteracted this favorable effect by increasing expression level of P16, IL-6 and MMP-13 while decreasing expression level of COL2A1 and ACAN (Fig. 6).

## Discussion

Although much progress has been made in the pathogenesis research and clinical treatment of OA, OA is still a major threaten to human health for elderly patients worldwide due to disability and pain of joints [24]. Over the past few years, metformin has received growing attention for its protective effects against OA and other age-related disease besides its hypoglycemic role. Metformin is reported to suppress IL-1β-induced oxidative and OA-like inflammatory changes by enhancing the SIRT3/PINK1/Parkin signaling pathway [25]. Administration of metformin gives a beneficial effect on long-term knee joint outcomes in obese OA patients by a reduced rate of medial cartilage volume loss and a reduction in risk of total knee replacement [26]. Therefore, revealing the roles of metformin and potential downstream genes of it is essential for elucidating the molecular mechanisms of OA and identifying new therapeutic targets for it.

Accumulating evidences show that microRNA-34a could be regulated by metformin [18, 19]. In this present study, the connection between metformin and microRNA-34a in OA was investigated and quantitative RT-PCR results showed that metformin treatment inhibited expression level of microRNA-34a in OA chondrocytes. However, conflicting results related with metformin and microRNA-34a have been reported. Several studies reported metformin could elevate microRNA-34a expression [27, 28], while more researchers demonstrated metformin could inhibit microRNA-34a expression [18, 19, 29], indicating that the interplay between metformin and microRNA-34a is more complicated than we thought and more efforts are needed to investigate the mechanisms of metformin on microRNA-34a. On the other hand, we found metformin incubation elevated SIRT1 expression in OA chondrocytes, which was also demonstrated by immunofluorescence staining.

It is known that microRNAs tend to exert their roles by regulating translation or stability of mRNAs of downstream target gene [13]. Specifically, microRNA-34a has been reported to directly target SIRT1 [30], which coincided with what we observed in this study that transfection of microRNA -34a mimic could inhibit SIRT1 expression in chondrocytes. Moreover, the siRNA we ordered could effectively inhibit SIRT1 expression, which is necessary for the following experiments. Thus, we hypothesized microRNA-34a and its well-studied target, the longevity-associated protein SIRT1, were involved in the biological function of metformin in OA.

Since aging is crucial to OA, any drug targeting cell senescence and associated chronic low-grade inflammation and metabolic disorder could be utilized to slow down OA progression. Metformin is thought to be an “anti-aging” drug, based on in vitro and animal experiments and numerous retrospective analyses on beneficial outcomes for type 2 diabetics and is being tested in several clinical trials evaluating the impact of metformin on aging [31–33]. Previous studies document microRNA-34a as an important regulator of age-dependent tissue changes and a cell senescence inducer in many diseases [34]. Meanwhile, SIRT1 has been reported to counteract multiple aging-associated diseases on account of its capacity to modulate a variety of cellular processes such as DNA repair, apoptosis, mitochondrial biogenesis and cell stress responses [35, 36]. Therefore, we assume that metformin ameliorates OA by regulating chondrocytes senescence through microRNA-34a/SIRT1 pathway. Firstly, we examined the effect of metformin on senescence in OA chondrocytes and results from SA-β-galactosidase staining suggest that metformin treatment attenuated chondrocytes senescence and overexpression of microRNA-34a or silencing SIRT1 by siRNA could reverse this effect. At the same time, we also detected that overexpression of microRNA-34a or silencing SIRT1 could counteract the stimulative effect of metformin on chondrocyte proliferation and cell clone formation. All these suggest metformin provided a protective effect to chondrocytes, while microRNA-34a/SIRT1 pathway is deeply involved.

Cellular senescence is a permanent state of cell cycle arrest and plays a significant role in the pathology of OA as OA chondrocytes shows elevated senescence-associated- β-galactosidase (SA-β-Gal) activity and accumulation of cyclin-dependent kinase (CDK) inhibitors P16, P21 [37]. Senescent chondrocytes exhibit inhibited proliferation capacity and loss of original phenotype, leading to imbalance of ECM metabolism [38]. In addition, OA chondrocytes undergo chronic and dynamic transformations into senescence-associated secretory phenotypes (SASP), characterized by increased secretion of proinflammatory cytokines, such as IL-1β, IL-6, TNF-α, and matrix metalloproteinases (MMP), such as MMP-13 [39]. In this study, we investigated the mechanisms of metformin on chondrocytes senescence. Western blot results demonstrated that metformin inhibited expression of P16, IL-6 and MMP-13 while elevated Collagen II and Aggrecan expression, indicating metformin ameliorated chondrocytes senescence through regulating P16 and attenuated OA by inhibiting SASP production and promoting extracellular matrix synthesis. Moreover, the restoration of microRNA-34a markedly counteracted this chondroprotective effect of metformin. Taken together, our study demonstrated that metformin inhibited chondrocyte senescence and promoted chondrocyte proliferation through microRNA-34a/SIRT1 pathway in osteoarthritis.

In summary, we suggest that metformin downregulated microRNA-34a and elevated SIRT1 in OA chondrocytes. Metformin ameliorated OA by inhibiting chondrocyte senescence and SASP secretion, as well as promoting chondrocyte proliferation and extracellular matrix synthesis via microRNA-34a/SIRT1 axis. These findings give us a new perspective of relationship between metformin and OA and could make metformin a novel choice for OA therapeutic strategy.

## Data Availability

All data generated or analyzed during this study are included in this published article.
